# Inflammation in HIV and Its Impact on Atherosclerotic Cardiovascular Disease

**DOI:** 10.1161/CIRCRESAHA.124.323891

**Published:** 2024-05-24

**Authors:** Laventa M. Obare, Tecla Temu, Simon A. Mallal, Celestine N. Wanjalla

**Affiliations:** Division of Infectious Diseases, Vanderbilt University Medical Center, Nashville, TN (L.M.O., S.A.M., C.N.W.).; Department of Pathology, Harvard Medical School, Boston, MA (T.T.).; Department of Pathology, Microbiology and Immunology, Vanderbilt University Medical Center, Nashville, TN (S.A.M.).; Department of Biomedical Informatics, Vanderbilt University Medical Center, Nashville, TN (S.A.M.).; Institute for Immunology and Infectious Diseases, Murdoch University, WA, Western Australia (S.A.M.).

**Keywords:** atherosclerosis, cardiovascular diseases, clinical relevance, endothelial cells, HIV, inflammation, risk factors

## Abstract

People living with HIV have a 1.5- to 2-fold increased risk of developing cardiovascular disease. Despite treatment with highly effective antiretroviral therapy, people living with HIV have chronic inflammation that makes them susceptible to multiple comorbidities. Several factors, including the HIV reservoir, coinfections, clonal hematopoiesis of indeterminate potential (CHIP), microbial translocation, and antiretroviral therapy, may contribute to the chronic state of inflammation. Within the innate immune system, macrophages harbor latent HIV and are among the prominent immune cells present in atheroma during the progression of atherosclerosis. They secrete inflammatory cytokines such as IL (interleukin)-6 and tumor necrosis-α that stimulate the expression of adhesion molecules on the endothelium. This leads to the recruitment of other immune cells, including cluster of differentiation (CD)8^+^ and CD4^+^ T cells, also present in early and late atheroma. As such, cells of the innate and adaptive immune systems contribute to both systemic inflammation and vascular inflammation. On a molecular level, HIV-1 primes the NLRP3 (NLR family pyrin domain containing 3) inflammasome, leading to an increased expression of IL-1β, which is important for cardiovascular outcomes. Moreover, activation of TLRs (toll-like receptors) by HIV, gut microbes, and substance abuse further activates the NLRP3 inflammasome pathway. Finally, HIV proteins such as Nef (negative regulatory factor) can inhibit cholesterol efflux in monocytes and macrophages through direct action on the cholesterol transporter ABCA1 (ATP-binding cassette transporter A1), which promotes the formation of foam cells and the progression of atherosclerotic plaque. Here, we summarize the stages of atherosclerosis in the context of HIV, highlighting the effects of HIV, coinfections, and antiretroviral therapy on cells of the innate and adaptive immune system and describe current and future interventions to reduce residual inflammation and improve cardiovascular outcomes among people living with HIV.

In recent decades, the landscape of HIV has undergone a transformative shift, attributed to remarkable advancements in antiretroviral therapy (ART). While these breakthroughs have significantly prolonged the lifespan of individuals living with HIV, a new set of challenges has emerged, particularly noncommunicable comorbidities such as cardiovascular disease (CVD).^1^ Traditionally perceived as primarily an immunodeficiency disorder, HIV is now recognized as a complex systemic condition that involves persistent immune activation, even in individuals effectively treated with ART.^2,3^ This sustained immune activation is associated with chronic inflammation, characterized by elevated levels of proinflammatory cytokines, immune cell activation, and heightened coagulation markers.^3,4^ The chronic immune activation and inflammation are major contributors to the pathogenesis of CVD in this population, which can persist even with effective ART.^5,6^ One mechanism for this persistent inflammation is viral persistence, where HIV can integrate into the genome of immune cells, creating a reservoir of virus that can persist for the lifetime of the infected individual.^3,7^ These infected cells can continue to produce viral proteins, leading to chronic immune activation and inflammation. Moreover, HIV proteins can directly activate endothelial cells with inflammation of the blood vessels.^8^ The combination of these factors can contribute to the development of atherosclerosis and other cardiovascular complications, implicating the chronic inflammatory state in the pathogenesis of various HIV-associated complications, including the acceleration of atherosclerotic cardiovascular disease (ASCVD).^2,9^

While we currently have no cure for HIV, the Berlin patient diagnosed with acute myeloid leukemia^10^ and the London patient diagnosed with Hodgkin lymphoma^11^ both achieved functional cure after allogeneic hematopoietic stem cell transplants with a homozygous CCR5 delta32 mutation. While the cancer treatments differed based on their diagnosis and responses, both patients had confirmed CCR5-tropic virus before transplant and did not experience HIV rebound after transplant.^11^ The London patient was noted to have cytomegalovirus (CMV) and Epstein-Barr virus reactivation 85 days after transplant requiring treatment.^11^ Notably, there were differences in the immune cell composition after transplant, the most significant being the lack of CCR5^+^ cluster of differentiation (CD)4 and CD8^+^ T cells. Inflated anti-CMV_pp65_ CD8^+^ pretransplant (8.5% of CD8 T cells) remained low after transplant (0.14%) at day 819, while anti-CMV CD4^+^ T cells, which at baseline are less clonal, went from 2% to 1.9% also at day 819 after transplant. There are more studies needed to understand the immune changes that happen after a functional cure for HIV, as we work toward a practical cure approach for the larger population who are not eligible for transplants.

Perturbations caused by HIV and its treatment can impact the drivers of CVD for both better or worse and are, therefore, of clinical relevance to people living with HIV (PLWH) and, more generally, to understand the pathogenesis of CVD in this population. These impacts have changed over the course of the HIV/AIDS epidemic (Table 1).

**Table 1.**
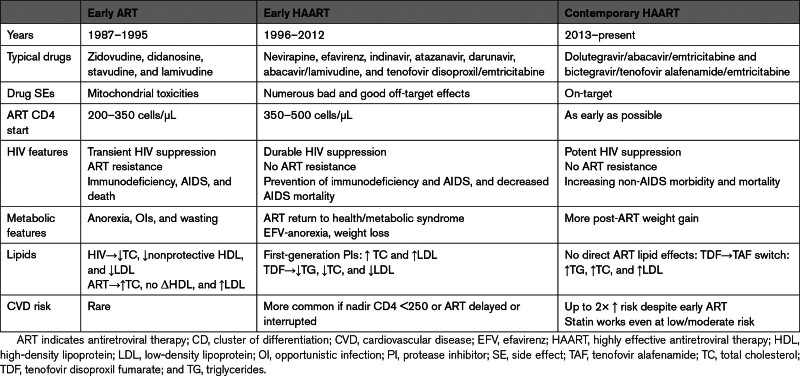
Changing Features of HIV Treatment and Outcomes in Different Eras of the Epidemic

Early ART (1985–1995) only transiently suppressed HIV replication in most patients until ART resistance, immunodeficiency, AIDS, and death almost inevitably occurred. Profound HIV-induced immunodeficiency was associated with the normalization of endothelial function, even in those with risk factors such as smoking and diabetes.^12^ During this era, most PLWH experienced anorexia and weight loss, and metabolic syndrome and CVD were rare.^13^ This pattern reversed in 1995 with the advent of highly effective ART, which sustainably inhibits HIV replication, preventing the emergence of resistance or immunodeficiency. While HAART was an advance in terms of prevention of AIDS and death, manifestations of metabolic syndrome more typical of the general population began to be seen.^14,15^

Despite earlier, more potent, and more on-target ART, PLWH still have up to twice the risk of CVD as those without HIV.^1,16^ The recently reported REPRIEVE study (Randomized Trial to Prevent Vascular Events in HIV) of 7769 PLWH with low-to-moderate risk of CVD reported a 35% reduction in major cardiovascular events in those randomized to pitavastatin compared with placebo.^17^ The findings are in keeping with an ongoing inflammatory response driving CVD in PLWH despite early and effective ART that may be mitigated by pitavastatin.

## ATHEROSCLEROSIS IN PLWH

Atherosclerosis is a complex chronic inflammatory disease characterized by endothelial cell injury, accumulation of lipids, immune cells, and fibrous elements within arterial walls. The progression of this process leads to the formation of coronary plaque with gradual narrowing and hardening of arteries, eventually resulting in ischemic cardiovascular events.^18^ PLWH are at a higher risk of developing ASCVD compared with the general population. The increased risk cannot be solely attributed to traditional CVD risk factors,^1,19–22^ as even PLWH with low CVD risk have significant coronary plaque burden associated with immune activation.^23^ Furthermore, PLWH may be up to 4.5× more likely to die from sudden cardiac death compared with controls.^24^ The pathophysiological mechanisms underlying this link are multifaceted, involving interactions between the immune system, viral persistence, and the endothelium. Among 1612 participants in the MACS (Multicenter AIDS Cohort Study) cohort, men living with HIV who have sex with men recruited from 4 different cities in the United States were shown have been shown to have prolonged QT intervals on 12-led standing resting electrocardiograms, which was associated with systemic inflammatory markers, including ICAM-1 (intercellular adhesion molecule 1), IL (interleukin)-6, and BAFF (B-cell activating factor).^25^

Decades of research have implicated cells of the innate and adaptive immune systems in the pathogenesis of ASCVD.^26^ HIV itself, when untreated, is a significant risk factor.^27,28^ However, it has also been shown that PLWH with AIDS are protected from ASCVD.^12^ Despite treatment with ART, HIV exerts enduring alterations in the memory pool of immune cells.^29^ These changes occur through various means, such as direct effects of the virus, coinfections, or ART.^7,30,31^ As such, PLWH have persistent systemic inflammation, which contributes to multiple comorbidities, including CVD.^2^ Early initiation of ART with acute HIV is associated with the near normalization of the coagulation cascade and numerous systemic inflammatory biomarkers. However, several inflammatory markers, including the acute-phase reactants, monocyte activation, and fibrosis biomarkers, remain elevated (Table 2).^5^

**Table 2.**
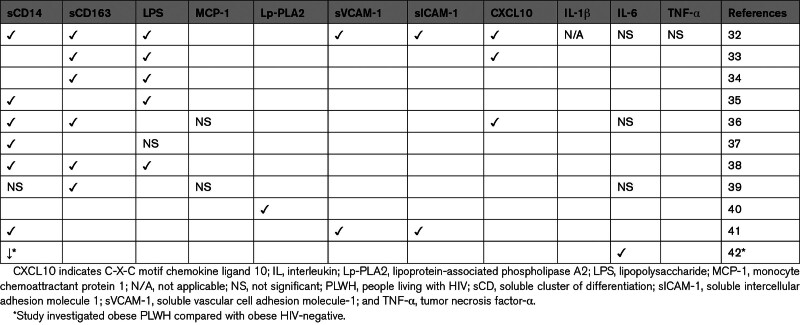
Elevated Inflammatory Markers in PLWH Compared With People Without HIV

**Figure 1.**
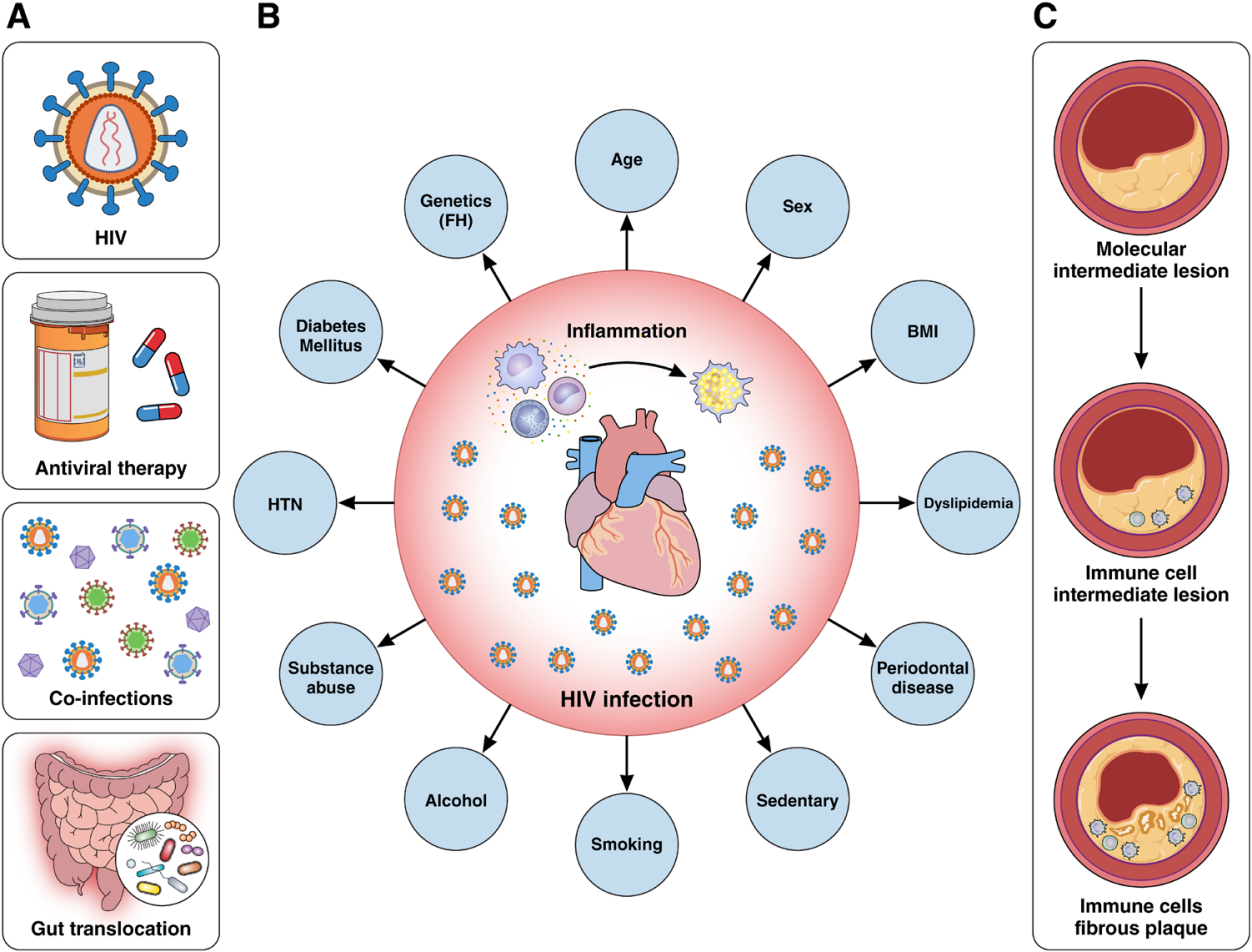
**Cardiovascular disease (CVD) risk factors in people living with HIV (PLWH) and people without HIV.** HIV-related risk factors (HIV infection, antiretroviral therapy, coinfections, and microbial translocation; **A**) and traditional risk factors (**B**) are major drivers of systemic inflammation that contribute to endothelial cell dysfunction and progression to atherosclerosis and CVD among PLWH (**C**). BMI indicates body mass index; CMV, cytomegalovirus; FH, family history; HCV, hepatitis C virus; and HTN, hypertension. Illustration credit: Sceyence Studios.

While monocytes are important in aging-related inflammation among PLWH and people without HIV, chronic infection with HIV is uniquely characterized by T-cell activation and exhaustion, as well as increased soluble inflammatory markers (such as sTNFR-1 [soluble tumor necrosis factor receptor 1], sTNFR-2, and IL-6) in ART recipients with undetectable viremia.^3,43–46^ Among PLWH, T-cell activation predicts short-term mortality and is associated with future cardiovascular events, emphasizing the potential impact on the broader health spectrum.^43,47^ In this review, we summarize the literature on the immune system’s role in CVD among PLWH. Specifically, we explore the role of cells of both the innate and adaptive immune systems and their impact on ASCVD progression. Furthermore, we discuss research gaps and summarize potential opportunities for future investigations to discover novel therapies (Figure 1).

### Endothelial Dysfunction

Endothelial dysfunction is an early precursor in the development of atherosclerosis.^48^ This manifests as impaired nitric oxide signaling with reduced ability for vascular relaxation. Injured endothelium allows the entry of cholesterol-rich lipoproteins, and ApoB (apolipoprotein B)–containing lipoproteins, into the subendothelial space. These lipoproteins undergo oxidative, enzymatic, and chemical modifications, activating endothelial cells and vascular smooth muscle cells.^49^ Activated endothelial cells increase the expression of adhesion molecules, including sVCAM-1 (soluble vascular cell adhesion molecule-1), sICAM-1 (soluble intercellular adhesion molecule 1), and MCP-1 (monocyte chemoattractant protein-1). The result is a chemokine gradient and increased expression of adhesion molecules that trigger a cascade of immune cell influx, dominated by innate and adaptive immune cells, particularly dendritic cells (DCs), monocytes/macrophages, and T cells.^50^

HIV induces endothelial dysfunction through direct and indirect mechanisms that are not fully defined. In vitro studies suggest a potential direct role for HIV replication, with viral proteins such as gp (glycoprotein) 120 and Tat (transactivator of transcription) exhibiting toxicity to cardiac and vascular cell lines.^51,52^ In vivo, HIV can infect some human endothelial cells (depending on the organ).^53^ In addition, HIV proteins, gp120, Tat, and Nef (negative regulatory factor), have been shown to stimulate endothelial cells to express inflammatory cytokines, such as IL-6, adhesion molecules (ICAM-1), and induce endothelial cell apoptosis. gp120 has also been associated with increased expression of MMP2/9 (matrix metalloproteinase 2/9) and IL-8.^8^ In addition to the direct effects of HIV, plasma cytokines and chemokines also stimulate the endothelium, leading to persistently elevated sVCAM-1 and sICAM-1 levels.^32,41,54–56^ Notably, sVCAM-1 levels decrease with ART.^6,57^ Despite the decrease in sVCAM-1 levels, PLWH on ART with effective viral suppression still have higher levels of endothelial inflammation compared with people without HIV. This is confounded by the data that implicate ART in endothelial dysfunction, adding complexity to the understanding of the underlying mechanisms in PLWH.^57,58(p1)^ Endothelial dysfunction among PLWH on therapy has also been linked to monocyte activation.^41^ In nonhuman primates, simian immunodeficiency virus (SIV) and simian-HIV infections were shown to cause an increase in endothelial proliferation, increased recruitment of subendothelial CD8+T cells and CD68+ macrophages/monocytes, and decreased endothelial function with decreased eNOS (endothelial nitric oxide synthase) compared with negative controls.^59^

Beyond peripheral endothelial dysfunction, HIV has also been associated with coronary microvascular dysfunction as measured by myocardial blood flow reserve derived from positron emission tomography.^60–62^ The pathogenesis of coronary microvascular dysfunction among PLWH is probably mediated by chronic inflammation; however, this is not fully understood.^61^ Like CVD, accumulating immune cells around pulmonary arteries is a risk factor for pulmonary arterial hypertension.^63^ HIV-related and non-HIV–related factors cause endothelial injury, pulmonary vasculopathy, and eventually HIV-associated pulmonary arterial hypertension.

### Fatty Streak Formation

With the accumulation of cholesterol-rich lipoproteins (Table 3), atherosclerotic plaques progress from intimal media thickening to early lesions, known as fatty streaks, characterized by cholesteryl ester-laden foam cells. This is followed by more advanced lesions containing a core region of activated immune cells, cholesterol crystals, and extracellular lipids.^73^ Elevated plasma cholesterol levels trigger the infiltration of cholesterol-containing lipoprotein particles into the artery wall. This infiltration is facilitated by interactions with proteoglycans of the extracellular matrix, prompting local cellular responses. Leukocyte adhesion molecules and chemokines expressed by vascular cells contribute to the recruitment of circulating monocytes into the tunica intima—the innermost layer of the artery.^50^

**Table 3.**
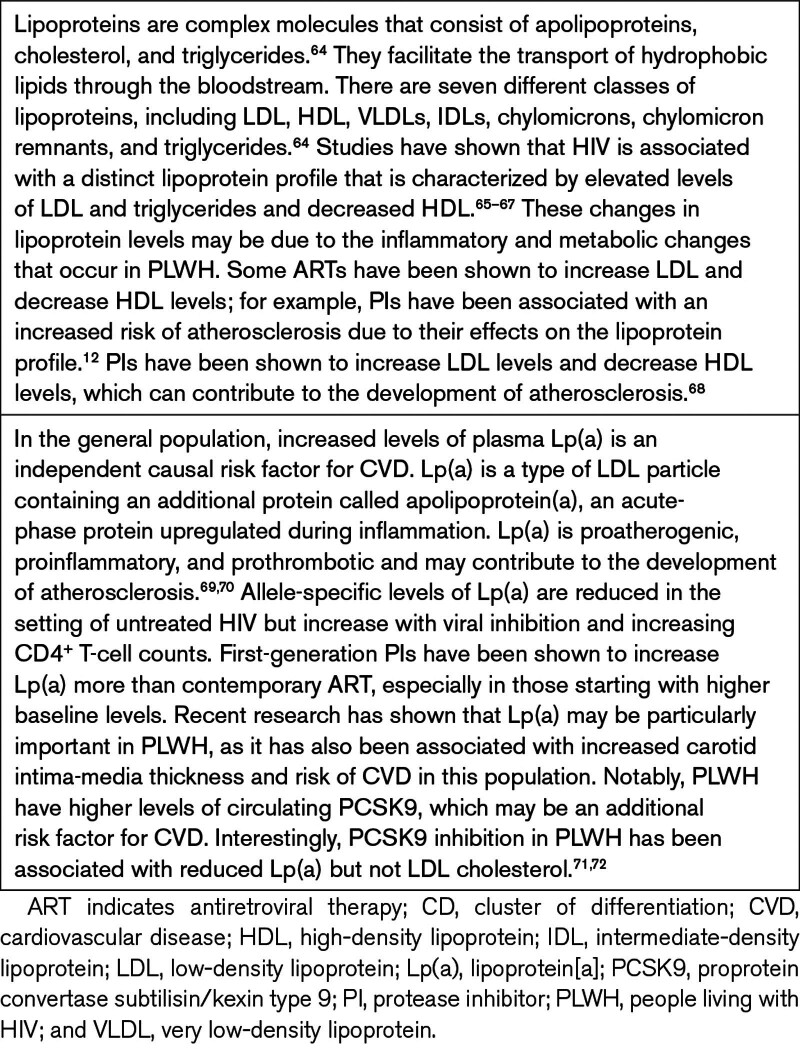
Plasma Lipoproteins in HIV and Atherosclerosis

Apart from its impact on the endothelium, HIV can also directly alter lipid metabolism.^65^ Immune activation in untreated PLWH may contribute to dyslipidemia due to direct effects of antiviral cytokines, such as IFN (interferon)-α, which are associated with increased serum triglycerides presumed to be from dysregulated clearance.^66,74^ In a more recent study, the MACS, in both untreated and treated PLWH, elevated IL-6, ICAM-1, and D-dimer levels were associated with decreased small HDL (high-density lipoprotein) particles.^75^ While treatment with the newer, highly active ART generally improves low HDL cholesterol, it does not fully normalize the lipid profile in treated PLWH. Notably, several classes of ART have also been implicated in altering lipid metabolism and increasing oxidative stress in PLWH, potentially independently contributing to an elevated cardiovascular risk.^76^

### Plaque Progression

Plaque progression is characterized by increased recruitment of inflammatory immune cells and alterations in the function of vascular smooth muscle cells. Vascular smooth muscle cells are traditionally known for their role in extracellular matrix production and exhibit phenotypic plasticity, including the acquisition of plaque-promoting macrophage–like features and plaque-stabilizing fibroblast–like features.^49^ Classical monocytes, recruited via CCR2 and CCL2 (C-C motif chemokine ligand 2), dominate the monocyte influx in atherosclerosis and differentiate into macrophages on stimulation by mediators such as M-CSF (monocyte-colony stimulating factor). As they transition, the macrophages initiate the uptake and clearance of lipoproteins, forming lipid-rich foam cells.^77,78^ Macrophages take up cholesterol through various mechanisms, leading to the accumulation of cholesteryl ester droplets in the cytosol and the formation of foam cells. Over time, these foam cells become overloaded, leading to cell death and the formation of a necrotic core within the atherosclerotic lesion.^79^ Consequently, this process triggers the release of proinflammatory cytokines and adhesion molecules crucial to plaque formation and growth. HIV may hinder the cholesterol efflux of monocytes, leading to enhanced foam cell formation due to altered expression or increased degradation of genes/proteins that regulate cholesterol efflux, such as cholesterol transporter ABCA1 (ATP-binding cassette transporter A1).^80,81^ The impaired cholesterol transport appears Nef dependent, which has been shown to directly bind to ABCA1, leading to downregulation and translocation of ABCA1 and accumulation of cholesterol in macrophages.^82^ A study of rhesus macaques infected with SIV also supports that Nef suppresses cholesterol efflux by ABCA1.^83^ SIV-infected macaques had lower expression of ABCA1 in the livers and coexpression of Nef, without evidence of replicating SIV.^83^ The accumulation of cholesterol in HIV-infected cells is thought to be important for HIV trans infection from monocytes, B cells, and DCs to CD4^+^ T cells. There are PLWH who are long-term nonprogressors where an aberrant lipid metabolism appears to be an important advantage that restricts trans infection of CD4+T cells from antigen-presenting cells.^84,85^

Macrophages within atherosclerotic plaques can display proinflammatory or anti-inflammatory features, with subsets such as inflammatory macrophages and type I IFN-inducible cells leading to plaque growth.^86^ Macrophage differentiation is associated with increased scavenger receptors, including SR-A (scavenger receptor class a), CD36, MARCO (macrophage receptor with collagenous structure), and LOX-1 (lectin-like oxidized LDL receptor 1). These pattern recognition receptors mediate the internalization of various molecules, including oxidatively modified lipoprotein particles.^86^ Several factors including netrin 1, produced in plaque macrophages, prevent emigration and lead to macrophage accumulation within the atherosclerotic lesion.^87^

### Noncalcified Versus Calcified Plaque

Several studies have shown that PLWH have a higher prevalence of noncalcified coronary plaque than calcified plaque.^23,36,38,88,89^ Previous studies in the general population suggest that unstable/vulnerable plaques have spotty calcification and a higher degree of inflammation.^90–92^ In PLWH, positive arterial remodeling in coronary arteries was seen with noncalcified plaque.^93^ There is limited literature that supports the role of monocyte activation in the development of noncalcified coronary plaque.^38^ In some studies, PLWH with noncalcified plaque were older and had higher sCD163.^36^ sCD163 is both a soluble and cell surface marker associated with atherosclerosis in people with and without HIV.^34^ While few studies point to the role of monocytes, the underlying mechanisms that promote noncalcified plaque in PLWH are not clear and need to be investigated further.

## THE ROLE OF THE INNATE AND ADAPTIVE IMMUNE SYSTEM IN ATHEROSCLEROSIS AMONG PLWH

**Figure 2.**
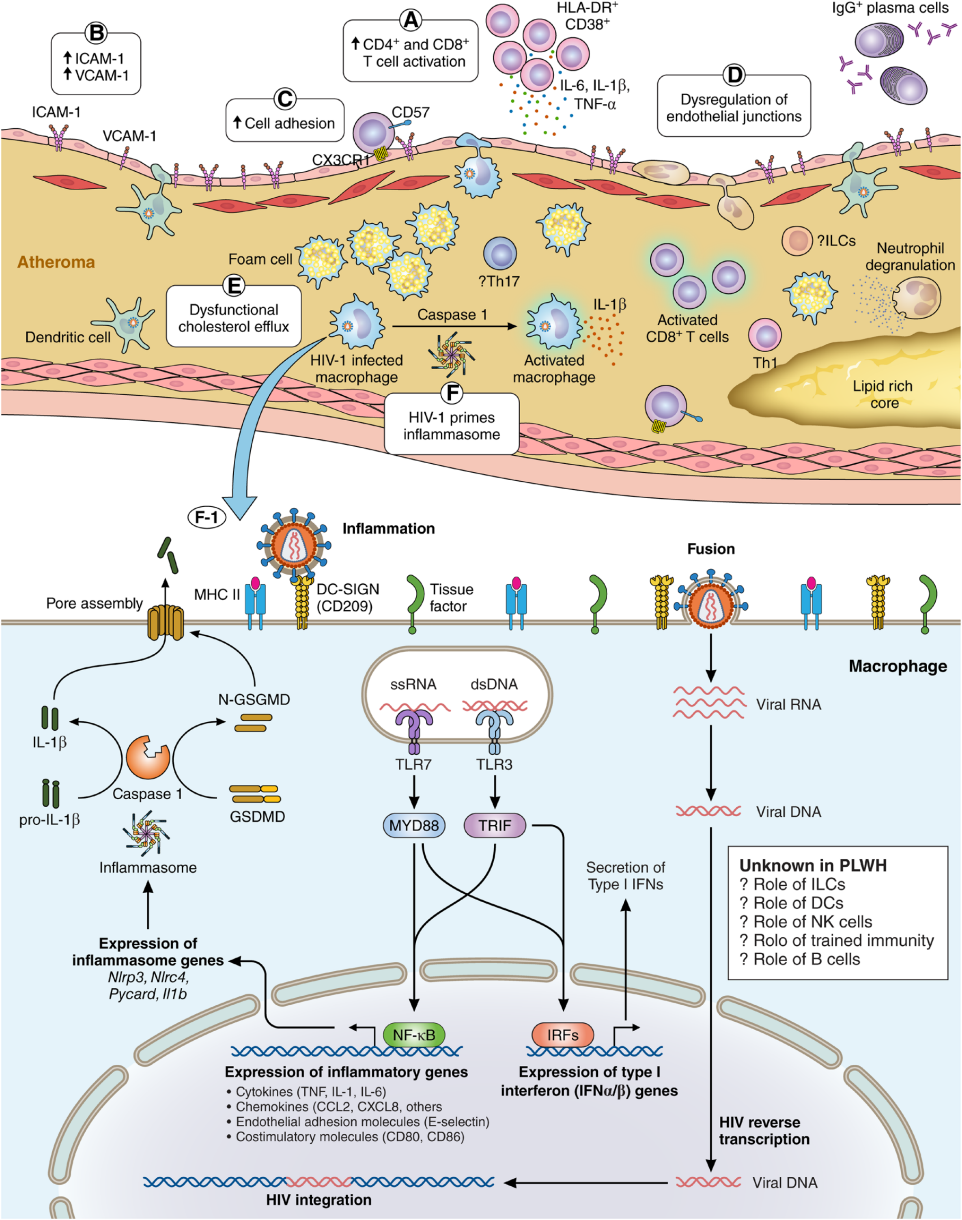
**Role of cells of the innate and adaptive immune system in atherosclerosis progression. A**, People living with HIV (PLWH) with viral suppression on antiretroviral therapy (ART) have increased CD4^+^ and CD8^+^ T-cell activation. **B**, The endothelial cell lining, due to multiple factors including viral replication and exposure to inflammatory cytokines, has increased expression of adhesion molecules, ICAM-1 (intercellular adhesion molecule 1), and VCAM-1 (vascular cell adhesion molecule 1). **C** and **D**, Cells of the innate and adaptive immune system adhere to inflamed endothelium and are recruited into the subendothelial space. **E**, Monocytes present in atheroma and infected with HIV are characterized by dysfunctional cholesterol efflux. **F** and **F-1**, HIV-1 primes the inflammasome and leads to activation of NF-κβ (nuclear factor kappa B), activation of caspase-1, and the expression of IL (interleukin)-1β. While the picture depicts plasma cells, innate lymphoid cells (ILCs), dendritic cells (DCs), and natural killer (NK) cells, they have not been studied among PLWH. CVD indicates cardiovascular disease; HLA-DR, human leukocyte antigen-DR; MHC, major histocompatibility complex; TLR, toll-like receptor; and TRIF, TIR-domain-containing adapter-inducing interferon-β. Illustration credit: Sceyence Studios.

Here, we summarize the changes in cells of the innate and adaptive immune response and their association with subclinical atherosclerosis (Figure 2).

## INNATE IMMUNE SYSTEM

### Monocytes/Macrophages

The central role of monocytes/macrophages in atherosclerosis is well-established in the general population. Of all the different monocytes, nonclassical monocytes are more permissive to HIV infection.^94^ Like other inflammatory disorders, PLWH not on treatment have significantly higher proportions of intermediate and nonclassical monocytes.^95(p14),96(p14)^ While ART reduces the level of inflammation observed in patients, longitudinal studies have shown that nonclassical monocytes remain elevated.^95^ In PLWH, several factors contribute to changes in the constitution of different monocyte subsets and their activation. In 2012, Funderburg et al showed that PLWH with and without treatment had higher proportions of both intermediate and nonclassical monocytes compared with people without HIV. Importantly, they showed that people without HIV with acute coronary syndrome and PLWH with viremia >400 had similar proportions of tissue factor (TF)^+^ monocytes. Furthermore, the increased expression of prothrombic markers (TF and CD62P) in PLWH appeared to be partly driven by both HIV and lipopolysaccharide.^97–99^ However, in a separate study with predominantly male participants on ART and undetectable, TF^+^ monocytes were not associated with carotid intima-media thickness (CIMT) as their CVD end point.^100^

Due to limitations in mechanistic studies with human study participants, biomarkers such as sCD163 and sCD14 have been heavily relied on as indicators of monocyte/macrophage activation and have been shown to be associated with multiple outcomes, including arterial inflammation, CIMT, and coronary plaque burden, among others (Table 4). Due to differences in study participants and known and unknown confounders, which include traditional CVD risk factors, the relationships between plasma biomarkers and cardiovascular outcomes are not always consistent.

**Table 4.**
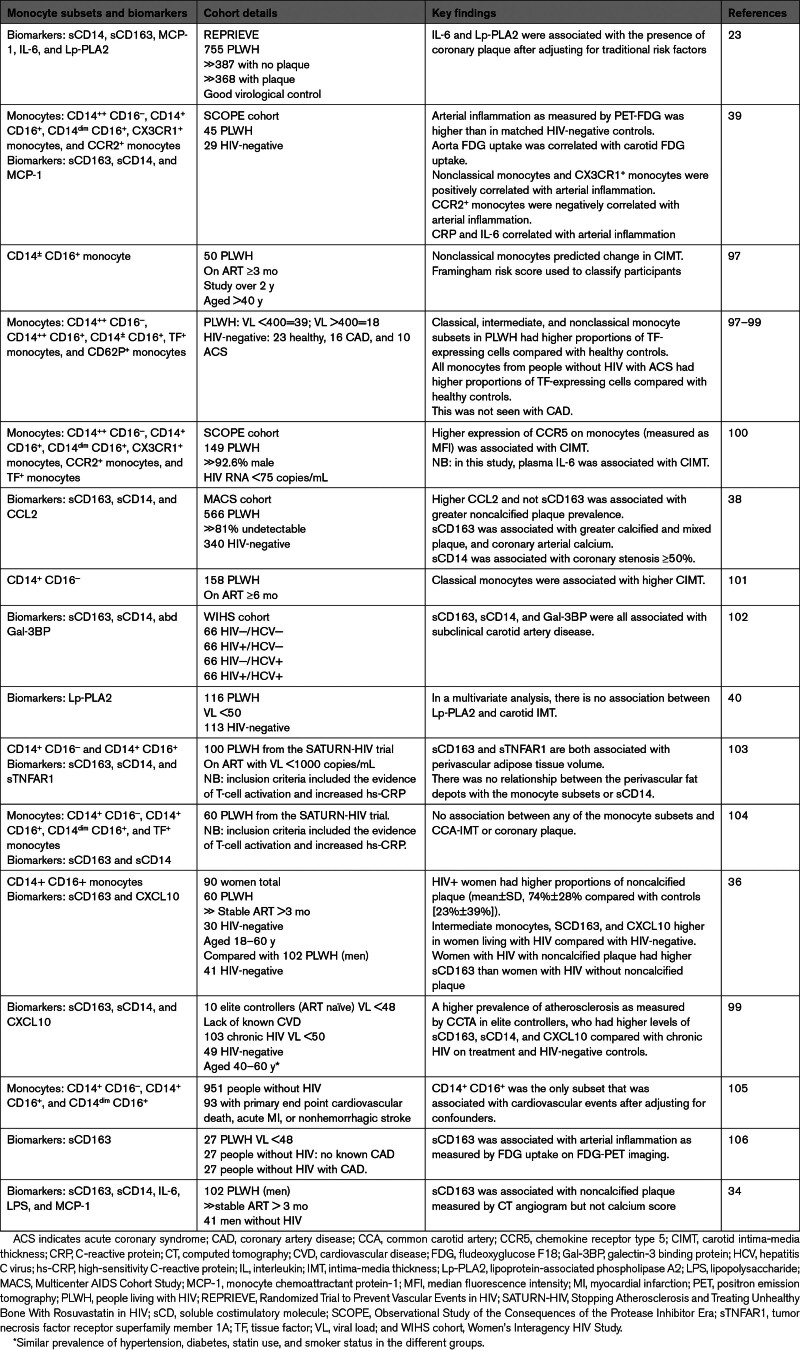
Role of Monocytes/Macrophages in CVD Among PLWH

Mouse models have provided different insights into the possible role of monocytes/macrophages in HIV-1–related atherosclerosis. HIV-1 Tg26 (transgene insertion 26) transgenic mice generated on an inbred laboratory mouse strain (FVB/N [friend virus B/NIH]) background were crossed with ApoE^−/−^ mice on the C57BL/6 background. The HIV-1 DNA introduced into these mice lacks the gag and pol genes with no productive HIV replication. However, these Tg26^+/−^/ApoE^−/−^ mice express HIV-1 transcripts in the bone marrow and thymus. Tg26^+/−^/ApoE^−/−^ mice developed accelerated atherosclerosis with a larger plaque area on a normal chow diet for 8 weeks compared with ApoE^−/−^ mice. HIV increased the caspase-1 activity observed in the Tg26^+/−^/ApoE^−/−^ mice with increased expression of the NLRP3 (NLR family pyrin domain containing 3) inflammasome and serum IL-1β (Table 5).^114^

**Table 5.**
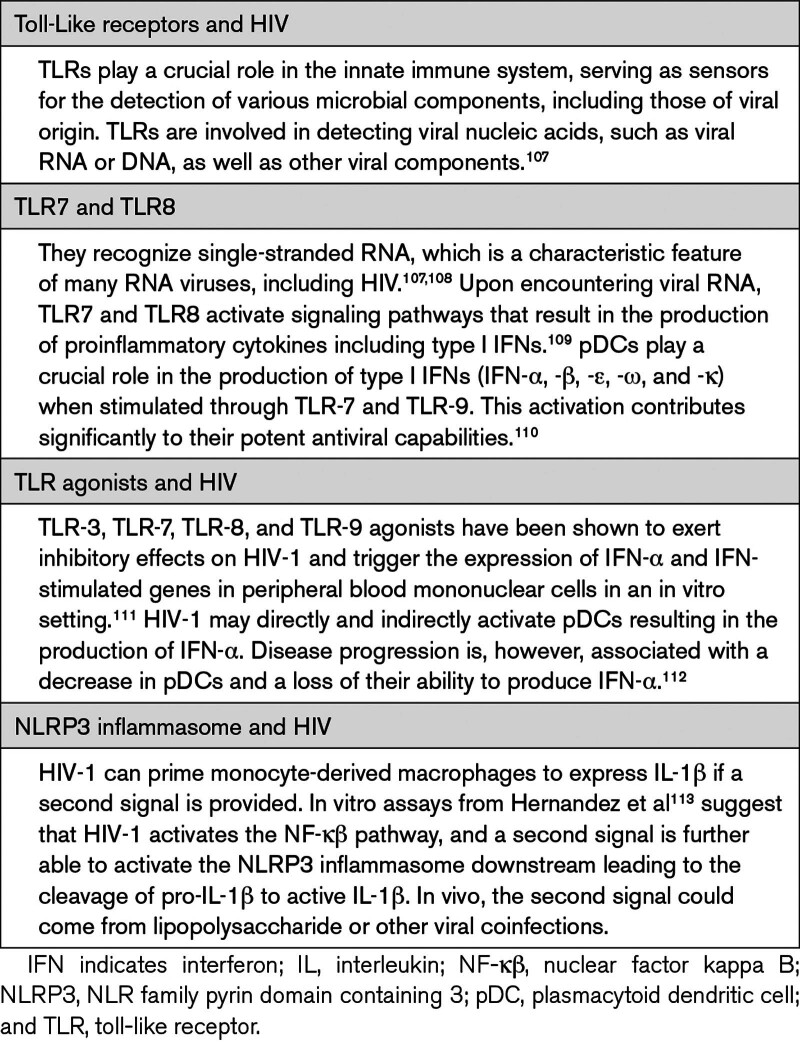
Innate Immune Receptors and HIV

Macaque models have also played a pivotal role in advancing our understanding of human diseases, including HIV and cardiometabolic diseases, offering a closer approximation to human physiology than mouse models.^115,116^ The ability of macaques to be infected with simian immunodeficiency virus, a close relative of HIV, provides a unique opportunity to study immune responses, viral replication, and disease progression in a system that more closely mimics infection in humans.^117^ In a study of macaques infected with SIV and allowed to develop acquired immune deficiency rapidly, increased monocyte and macrophage activation appeared to be linked to the codevelopment of encephalitis and cardiac inflammation in macaques. In brief, the infected macaques that developed both SIV-related encephalitis and CVD had higher levels of plasma sCD163 and IL-18 and had a higher proportion of cardiac macrophages (CD68^+^, CD163^+^, CD206^+^, and MAC387^+^). There were no differences in total CD3^+^ T cells within the tissue.^118^ Notable, PLWH with HIV encephalitis were similarly found to have increased cardiac inflammation and fibrosis with more CD68+, CD163+, and MAC387^+^.

### Monocytes and CHIP

CHIP is characterized by mutations in genes such as *DNMT3A*, *TET2*, *JAK2*, and *ASXL1*. These mutations confer a selective advantage to hematopoietic cells, leading to clonal expansion, and have been associated with an increased risk of atherosclerotic CVD due to its proinflammatory effects.^119–123^ Murine models have previously shown that accelerated development of atherosclerosis is an essential factor contributing to elevated cardiovascular mortality in the presence of clonal hematopoiesis. This acceleration is attributed to enhanced expression of IL-6/IL-1β and stimulation of the NLRP3 inflammasome–triggered endothelial damage. The enhanced inflammatory interactions between the endothelium and macrophages originate from circulating clonal monocytes.^119,120,123^ In hyperlipidemic mice, CHIP increases IL-6/IL-1β expression and the progression of atherosclerosis.^124^

While CHIP is commonly associated with aging, its presence in individuals with HIV introduces a unique dimension, potentially intensifying the proinflammatory environment inherent to HIV.^125,126^ Bick et al^125^ demonstrated a 2-fold increase in the prevalence of CHIP among PLWH with increased mutations of the *ASXL1* gene. In this study, HIV was the second strongest factor, after age, linked to elevated CHIP prevalence among PLWH.^125^ These findings were consistent with findings from an Australian cohort where age was related to increased prevalence of CHIP among PLWH. Although mutations were observed in *DNMT3A* and *TET2*, the greatest mutations were reported in *ASXL1* in this population. This cohort also reported an increase in the inflammatory biomarker IL-6.^126^ Notably, among PLWH from the REPRIEVE trial, participants from sub-Saharan Africa, South Asia, and Latin America/Caribbean had lower odds of CHIP. Smoking and age were associated with increased odds of CHIP.^127^ The ongoing inflammatory conditions in HIV may foster clonal dominance of specific mutated hematopoietic stem cells, potentially explaining the higher occurrence of clonal hematopoiesis in individuals with HIV. Furthermore, the weakened immune response during acute HIV or the persistent inflammation and immune activation in chronic HIV could impede the body’s ability to survey and eliminate these clonal hematopoietic stem cell populations, further contributing to the elevated prevalence of CHIP in PLWH.^126^ The chronic immune activation and inflammation associated with HIV, combined with the potential inflammatory effects of CHIP, may potentiate CVD risk in this population.

### Dendritic Cells

DCs play a crucial role in bridging the innate and adaptive immune responses through their function of antigen capturing and presentation.^128^ DCs can be classified as conventional DCs/myeloid DCs and plasmacytoid DCs (pDCs) in blood and Langerhans cells in tissues.^129^ Each of these subsets has different phenotypes and functions.^130^ pDCs have proinflammatory roles as they produce ≈95% of type 1 IFNs in peripheral blood mononuclear cells in response to infection. Under noninflammatory conditions, DCs from healthy arteries likely present self-antigens, leading to T-cell tolerance. However, in atherosclerotic lesions, the maturation of plaque DCs in the presence of TLR (toll-like receptor) agonists, danger-associated ligands, and proinflammatory cytokines favors a proatherogenic activation status.^131,132^

In vitro studies show that conventional DCs and pDCs are both susceptible to HIV. HIV infects DCs, facilitating viral spread to memory CD4^+^ T cells.^129,133^ Conventional DCs from PLWH exhibit reduced levels and compromised function, which ART does not restore. HIV-1 may directly and indirectly activate pDCs, resulting in the production of IFN-α. The recognition of HIV ssRNA by TLR7 in pDCs activates the MYD88 pathway, stimulating IRF7 and IFN-α secretion (Table 5).^112^ Disease progression is, however, associated with a decrease in pDCs and a loss of their ability to produce IFN-α.^112^ While IFN-α controls virus spread, its continuous release can trigger immunosuppressive pathways, immunopathogenesis, and immunodeficient syndromes. The alternative TLR7-NF-κβ (nuclear factor kappa B) pathway also induces type I IFN production, although in lower amounts than lipopolysaccharide-stimulated DCs.^134^

It is important to note that, to our knowledge, no direct link has been established in the role of DCs in the progression of atherosclerosis in PLWH. However, HIV infection of DCs and subsequent activation may be important in atherosclerosis progression (Table 6). Further studies are needed to explore this potential connection.

**Table 6.**
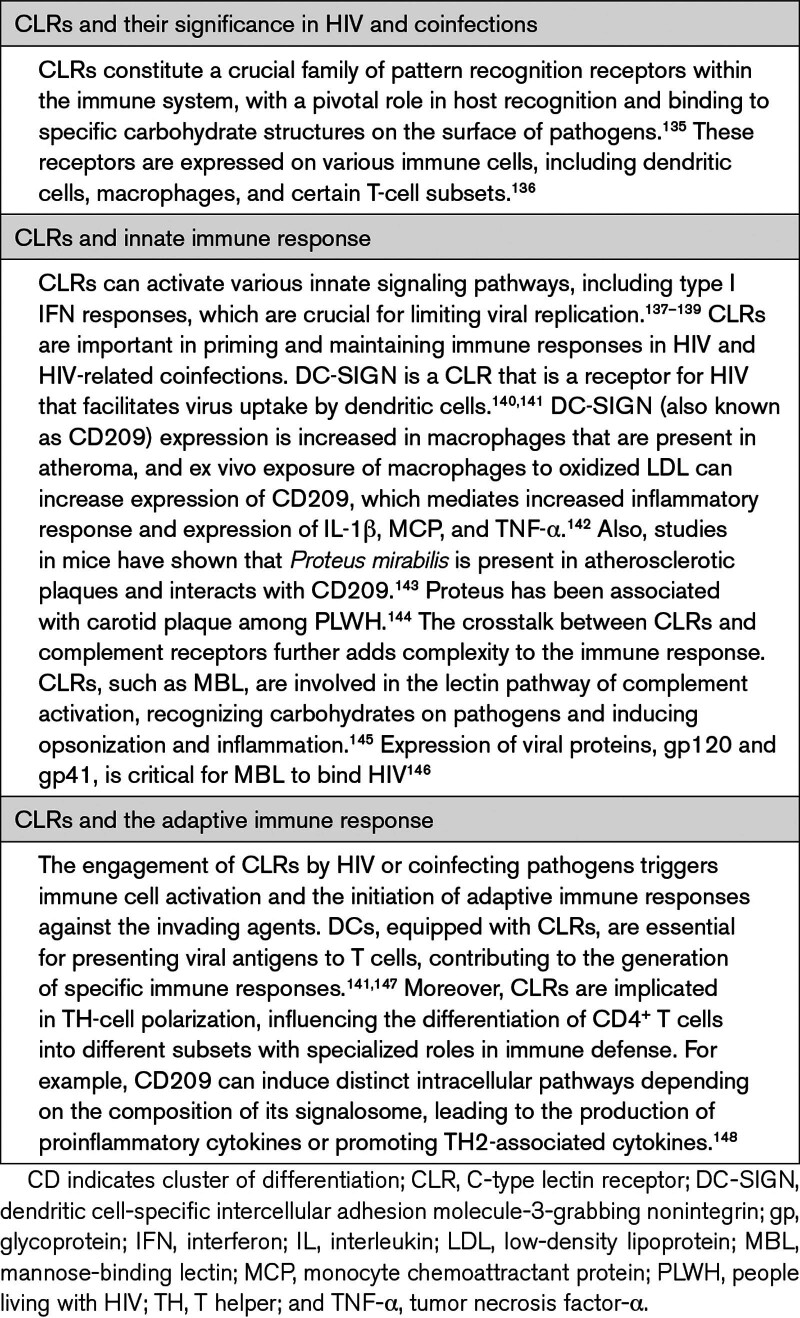
C-Type Lectin Receptor and Their Significance in HIV and Coinfections

### Hypercoagulability

HIV induces a chronic inflammatory response, leading to endothelial dysfunction and activation of the coagulation cascade.^149^ The virus may also directly infect endothelial cells, further contributing to a prothrombotic state. Activated endothelial cells may promote platelet activation, inflammatory reactions, and progression of atherosclerosis.^150,151^ As discussed in previous sections, persistent inflammation due to chronic HIV can lead to increased levels of proinflammatory cytokines, such as IL-6 and hs-CRP (high-sensitivity C-reactive protein), and other mediators, which, in turn, activate the coagulation system and increase the risk of CVD.^2,5,9,150^ D-dimer, a byproduct of the coagulation cascade and a marker for inflammation, is elevated in HIV. Research has indicated that it shares comparable associations with CVD and all-cause mortality, such as IL-6 and hs-CRP.^4,150,152^

Increased levels of circulating D-dimer, minor rises in blood coagulation, thrombin formation, and the turnover of cross-linked intravascular fibrin in PLWH suggest potential relevance to coronary heart disease and peripheral atherosclerosis.^153^ Additionally, D-dimer values could serve as indicators of inflammatory states.^154^ In a more recent study, the MACS study, in both untreated and treated PLW, elevated IL-6, ICAM-1, and D-dimer levels were associated with decreased small HDL particle number.^75^ Another subanalysis of the Strategic Management of ART (Antiretroviral Therapy) (SMART) cohort reported an increased risk of fatal CVD events with an increase in D-dimer score.^152^ The significant association of baseline D-dimer with the severity of CVD outcomes may reflect the activation of coagulation systems in response to low-grade inflammation associated with HIV infection.

Additionally, HIV has been associated with various changes in coagulation factors, such as an increase in procoagulant factors such as anti-phospholipid antibodies, a decrease in anticoagulant factors such as deficiencies in protein C or S, and heightened platelet activation.^155–159^ Some of these changes are similar to those seen in acute coronary syndrome.^160^ HIV and immune activation may also enhance the expression of TF on endothelial cells or leukocytes, thereby activating the extrinsic coagulation pathway.^161,162^ Previous studies have revealed higher TF expression on circulating blood mononuclear cells in PLWH than in uninfected controls. This study observed correlations between TF expression, HIV RNA levels, the proportion of CD8^+^ T cells expressing immune activation markers, and D-dimer levels.^162^ Based on their findings, direct activation of monocytes by microbial products (eg, via bacterial translocation from the gut) may be a key player in promoting thrombosis in HIV.

### Innate Lymphoid Cells and Unconventional T Cells

Innate lymphoid cells (ILCs) include the noncytotoxic group 1 (ILC1), group 2 (ILC2), and group 3 (ILC3) and cytotoxic-like conventional natural killer cells.^163,164^ These cells are characterized by their lymphoid morphology, inability to undergo clonal expansion when stimulated and lack of antigen-specific receptors such as the BCR (B-cell receptor) and TCR (T-cell receptor).^165,166^ Recent studies have implicated ILCs, especially ILC1s and ILC3s, in modulating the inflammatory milieu within atherosclerotic plaques. ILCs can express proinflammatory cytokines, such as IFN-γ and IL-17A, thereby influencing the recruitment and activation of other immune cells in the atherosclerotic microenvironment.^167^ Some studies suggest a protective function, and others propose a pathogenic role through the production of proinflammatory cytokines such as IFN-γ and IL-17A.^168^ Vu et al^168^ demonstrated that in ApoE^−/−^ mice, IL-17A may contribute to the proatherogenic role of γδ T cells, as many aortic γδ T cells in this study expressed IL-17A, but not IFN-γ.^113^ γδ T cells are abundant between the intima and atherosclerotic plaque and constitute about 20% of the T-cell population.^169^ These cells can recognize stress-induced antigens within atherosclerotic plaques in the early stages.

ILC1s contribute to the inflammatory cascade by activating macrophages and DCs. This activation fosters the formation of foam cells and exacerbates endothelial dysfunction, ultimately promoting plaque destabilization.^170^ On the other hand, ILC3s, with their production of IL-17A and IL-22, further intensify the inflammatory environment within arterial walls. IL-17 promotes the recruitment of neutrophils and macrophages, while IL-22 exhibits both protective and pathogenic effects.^171(p3)^

In atherosclerotic-prone murine populations, ILC2 expansion has been suggested to have an atheroprotective role. Results from one study highlighted that ILC2-derived IL-5 and IL-13 are important contributors to this regulatory mechanism.^172^ The atheroprotective effects of IL-5 were confined to the thoracic aorta, and the production of IL-5 and IL-13 by other cell types failed to compensate for the absence of these ILC2-derived cytokines. Furthermore, it was observed that IL-5–dependent atheroprotection could not be attributed to alterations in macrophage phenotype or B cells (B1-dependent) natural IgM production. IL-13–dependent atheroprotection was linked to significant changes in collagen deposition and macrophage phenotype, suggesting a potential involvement of alternative activation pathways. However, the direct connections between changes in macrophage phenotype and atheroprotection were not explicitly addressed in the study.^172^

Among PLWH, a study by Kløverpris et al^173^ showed that all ILCs (ILC1-3) were depleted from the peripheral blood at the time of acute infection, with the transcriptional analysis pointing to death due to a strong response to IFNγ and gut inflammation. If ART was initiated at the time of the acute infection, then there was preservation of ILC. Otherwise, the ILCs were irreversibly depleted. Notably, they found that the remaining ILCs were activated and did not migrate into tissues.

### Trained Innate Immunity and HIV

PLWH have changes in cells of the innate immune system consistent with reprogramming, likely due to constant exposure to HIV and chronic inflammation from HIV. Specifically, one study found that monocytes from PLWH expressed higher levels of IL-1β when exposed to certain stimuli ex vivo (eg, *E coli* lipopolysaccharide and imiquimod). However, this increased sensitivity was not seen with lymphocyte activation.^174^ Trained immunity involves both transcriptional and metabolic reprogramming of innate immune cells. In addition to myeloid cells, other cells can develop trained immunity, such as natural killer cells (by CMV), ILCs, and stromal and epithelial stem cells.^175^ Many reviews synthesizing the literature on infections and CVD risk propose the role of trained immunity in atherosclerosis. Further research is needed in this area as new therapeutic targets are studied.^176^

## ADAPTIVE IMMUNE SYSTEM

T cells and B cells, present in both the adventitia of healthy arteries and atherosclerotic lesions, are recruited to atheroma by cells of the innate immune system and play a crucial role in the pathogenesis of this chronic inflammatory disease.^177,178^ Chemokine receptors, such as CCR5 and CXCR6, mediate the infiltration of T cells into plaques, with CCL5 and CXCL16 serving as key chemotactic signals. Notably, blocking the CCL5-CCR5 interaction leads to reduced infiltration of effector CD4^+^ T cells into aorta explants, underscoring the importance of these interactions in atherosclerosis progression.^179,180^

### T Cells

T cells are present in all stages of atheroma progression, and clonal T cells with oligoclonal TCRs are enriched in unstable plaques.^181,182^ The presence of oligoclonal TCRs suggests that T-cell expansion is antigen-driven and not stochastic. Antigens that have been implicated in CVD include autoantigens, such as oxidized LDL (low-density lipoprotein),^183^ ApoB, the core protein of LDL,^184^ heat-shock protein 60,^185^ and microbial-derived antigens from pathogens such as CMV.^186^ Blocking of T-cell activation using antibodies against CD40 ligand reduced the lipid content and size of atherosclerotic lesions and the numbers of T cells and macrophages present in the atheroma.^187^ Mechanistic studies in mice suggest a role for both CD4^136,137^ and CD8 T cells in accelerating atherosclerosis.^188,189^

CD4^+^ T-cell subpopulations (T helper [TH] 1,^190^ TH2,^191^ TH17,^192^ CD4^+^ T regulatory [Treg] cells, and cytotoxic T cells) are present in atheroma and influence the different stages of atherosclerosis. TH1 cells, characterized by the expression of the transcription factor T-bet, contribute to plaque development and instability through the secretion of IFN-γ.^193^ TH2 cells, expressing cytokines such as IL-4, IL-5, and IL-13, exhibit varying effects on atherosclerosis. IL-5 induces the production of oxLDL (oxidized low-density lipoprotein)-targeting IgM antibodies, contributing to antibody-dependent clearance of apoptotic cells and dampening inflammation. On the other hand, IL-13 deficiency accelerates atherosclerosis progression, while low-dose IL-13 stabilizes existing plaques through various mechanisms.^179^ The role of TH17 cells remains controversial, with evidence supporting both proatherogenic and plaque-stabilizing effects.^184,194,195^ T follicular helper cells, known for their role in humoral immunity, are implicated in atherosclerosis, particularly in regulating antibody isotype switching and affinity maturation.^196^ Conversely, Tregs, including FOXP3^+^ (forkhead box P3) Treg cells and IL-10^+^ type 1 regulatory T cells, are thought to exert an antiatherogenic.^197^ Treg cell depletion is associated with increased lesion size and proatherogenic shifts in the plasma cholesterol profile, emphasizing their regulatory role in vascular inflammation.^18,180^ Studies on atherosclerosis regression models indicate that the restoration of plaque Treg cell numbers is associated with an anti-inflammatory microenvironment, supporting tissue repair and dampening inflammation through enhanced efferocytosis and proresolving lipid mediators.^131^

Among PWLH, several studies suggest a role for T cells in the pathogenesis of CVD. HIV primarily infects CD4^+^ T cells, weakening the immune defenses of the host.^198^ PLWH with untreated and treated HIVs have higher levels of activation of both CD4+ and CD8+T cells, characterized by CD38 and HLA-DR coexpression.^43,44,199,200^ A study on PLWH on ART (viral load <400 copies/mL) and on rosuvastatin with fasting LDL cholesterol <130 mg/dL showed an association between activated CD8^+^ T cells (CD38^+^ HLA-DR^+^) and common carotid artery-intima media thickness (CCA-IMT).^104^ Other studies have also shown an association between CD8^+^ T-cell activation in PLWH on ART with virological suppression,^47,201,202^ CD4^+^ T-cell activation,^199,200^ and T-cell senescence (CD4 and CD8) with subclinical atherosclerosis.^47,199,201^ In a large study from the Veterans Aging Cohort Study, atherosclerotic CVD was associated with CD4^+^ T cells (T effector memory expressing RA) and TH17 cells. Incident CVD, on the other hand, has been associated with CD45RA+ CD4+T cells (T effector memory expressing RA), senescent cells, and TH17 cells.^203^ Notably, this study found an increased association between CD4^+^ Treg and the ASCVD risk, which could have been a compensatory increase with systemic activation.^203^

However, another notable difference in T cells by HIV is the inflation of CMV-specific CD4^+^ T cells, which is not evident in transplant patients and appears specific to PLWH. The inflation of anti-CMV–specific CD4^+^ T cells persists in PLWH on ART and may be driven by the patient’s HLA type.^29^ CMV-specific T cells in PLWH are associated with subclinical atherosclerosis.^204^ Furthermore, they express high levels of inflammatory markers (IFN-γ and TNF-α [tumor necrosis factor-α]) and can induce the expression of CX3CL1 (C-X3-C motif chemokine receptor 1) on the endothelium.^205^ In a study done in rhesus macaques and older PLWH, they found increased CX3CL1 and IL-15 expression, which was necessary for the recruitment of CX3CR1^+^ CD8^+^ T cells into atherosclerotic plaques.^206^ Several studies have shown an association between senescent T cells (CD28- CD8^+^ T cells) and subclinical atherosclerosis.^207^ A subset of CX3CR1^+^ CD4^+^ T cells coexpressing markers related to late activation/senescence (CD57) and cell adhesion (GPR56) is also associated with subclinical atherosclerosis, and immunohistochemistry of coronary arteries placed these cells within the adventitia and perivascular adipose tissue.^208^ Notably, late fibroatheroma from PLWH donors has been shown to have a lower percentage of CD4^+^ T cells in the coronary artery compared with HIV-negative.^208^ A relatively large cross-sectional study that combined male and female participants from the WIHS and MACS study found that among 1931 PLWH compared with 859 people without HIV, having a low CD4^+^ T-cell count was associated with a higher risk of developing subclinical atherosclerosis (1.74× higher in male participants and twice the risk in female participants).^209^

### B Cells

B cells exhibit various functions that might modulate the immune response in atherosclerosis, including antigen presentation, cytokine production, and lymphoid-tissue organization.^210^ Unlike T cells, B cells exhibit a distinct distribution within atherosclerotic lesions. While only few B cells are detectable locally within the atheroma, a significant number is found in the adventitial layer of atherosclerotic vessels, forming structures resembling tertiary lymphoid organs.^211^ These B cells within atherosclerotic lesions display oligoclonality and undergo antigen-driven proliferation, indicating a Th-cell–dependent, delayed immune response resulting in high-affinity antibodies, including IgM, IgG, IgE, and IgA.^212^

The role of B cells in atherosclerosis remains controversial, with some subsets being atheroprotective while others being atherogenic.^213,214^ For example, follicular B cells may exacerbate atherogenesis by initiating germinal center responses and producing IgG antibodies aimed at oxLDL, a process associated with CVD.^215^ Furthermore, these follicular B cells participate in the creation of tertiary lymphoid organs in the tunica adventitia of blood vessels, which are also impacted by atherosclerosis, leading to the formation of germinal center and in situ production of anti-oxLDL IgG.^216,217^

Marginal zone (MZ) B cells, on the other hand, are thought to be atheroprotective, as they inhibit the function of follicular helper T cells in the germinal center through PD-1 (programmed death-1)/PD-L1 (programmed death-ligand 1) interactions.^218,219^ Interestingly, this protective function depends on the expression of NR4A1 (nuclear receptor subfamily 4 group A), with its deletion in mice exacerbating the development of atherosclerosis due to a reduction in PD-L1.^220^ Furthermore, unlike IgG, anti-oxLDL IgM is seen as protective against atherosclerosis.^214^ Given that MZ B cells are significant producers of IgM,^215,221^ this could be linked to their protective role against atherosclerosis.

B-cell dysfunction and dysregulation have been well reported in HIV,^222–224^ and this has been associated with the progression of HIV. However, few studies have demonstrated the role of B cells in the development and progression of atherosclerosis in PLWH. Targeting BAFF has previously been shown to reduce the progression of atherosclerosis.^225^ BAFF is a cytokine involved in the selection and activation of the MZ population and is also important in B-cell survival and differentiation of innate cells.^226^ BAFF is elevated in PLWH and is correlated with viral factors, Nef, and markers associated with inflammation.^227^ A Montreal cohort involving PLWH on ART for 15 years reported elevated levels of BAFF in this population. At the baseline, low expression of BAFF is associated with higher expression of Breg markers on MZ B cells. However, PLWH on ART 15 years had reduced expression of Breg markers on a subset of MZ B cells associated with subclinical atherosclerosis.^228^

## OXIDATIVE STRESS AND LIPID OXIDATION

Oxidative stress occurs when there is an imbalance between the production of reactive oxygen species and the body’s ability to neutralize them with antioxidants.^229^ This imbalance can lead to the oxidation of lipids, including LDL cholesterol, a major contributor to the development of ASCVD. In HIV, oxidative stress is increased due to chronic inflammation and immune activation, which leads to the production of reactive oxygen species by activated immune cells.^230^ These reactive oxygen species can damage lipids, proteins, and DNA, leading to a wide range of cellular dysfunction.^230^ Additionally, ART has been shown to increase oxidative stress and lipid peroxidation. This is thought to be due to the interaction between ART and mitochondrial function, which can lead to the production of reactive oxygen species and cellular damage.

Lipid oxidation, specifically the oxidation of LDL cholesterol, has been implicated in the development of ASCVD in HIV. Oxidized LDL cholesterol is taken up by macrophages in the arterial wall, leading to the formation of foam cells and the initiation of the atherosclerotic process.^231^ In HIV, oxidized LDL cholesterol levels are increased, and this is thought to be due to the combination of chronic inflammation, immune activation, and ART-induced oxidative stress.^232^

## GUT TRANSLOCATION

The gastrointestinal tract experiences significant CD4^+^ T-cell depletion throughout all phases of HIV disease and is a significant HIV reservoir.^233^ This reduction predominantly affects the CCR5^+^ CD4^+^ T-cell subset, constituting the majority of CD4^+^ T cells in the gastrointestinal tract.^234^ A loss of CD4^+^ T cells in the gastrointestinal tract disrupts the delicate balance of the intestinal mucosal barrier with increased gut permeability, facilitating the translocation of microbial products, such as bacterial lipopolysaccharide, into the systemic circulation.^35,235^ Microbial translocation has been linked to systemic inflammation because the presence of microbial products in the bloodstream activates immune cells, initiating a chronic inflammatory state.^236^ In PLWH, a low CD4^+^ T-cell count is associated with higher plasma lipopolysaccharide.^32^

## POSSIBLE ROLE OF COINFECTIONS

**Figure 3.**
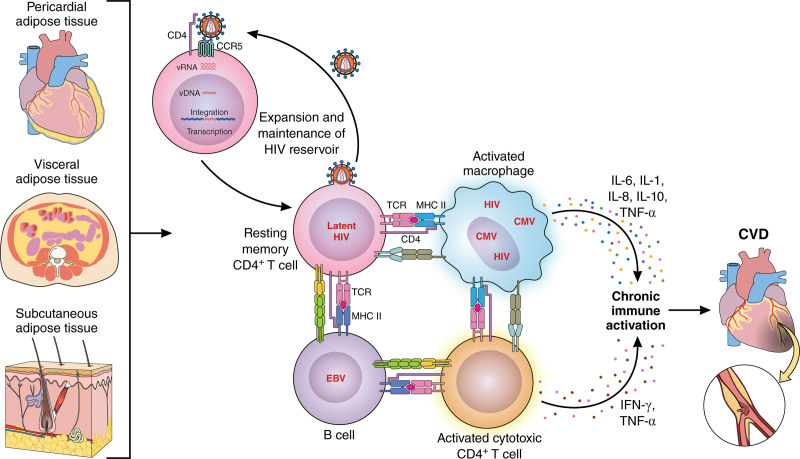
**Proposed model of how HIV and other chronic persistent viruses maintain the HIV reservoir and drive chronic inflammation contributing to cardiovascular disease (CVD).** HIV-1 reservoir cells consist of activated cluster of differentiation (CD)4^+^ T cells (predominantly cytotoxic, such as CGC^+^)^218,219^ that are resistant to killing by CD8^+^ T cells and natural killer (NK) cells.^238^ CD4^+^ T cells specific for Epstein-Barr virus (EBV) are stimulated by memory B cells that constitutively express latent EBV proteins and present them on MHC class II. Cytomegalovirus (CMV) expression by activated macrophages and endothelial cells can also activate T cells that express TCRs (T-cell receptors) that bind CMV epitopes, leading to the secretion of inflammatory cytokines that perpetuate the state of chronic immune activation. CCR5 indicates chemokine receptor type 5; IL, interleukin; IFN, interferon; MHC, major histocompatibility complex; and TNF-α, tumor necrosis factor-alpha. Illustration credit: Sceyence Studios.

Chronic HIV stands out among infectious diseases due to its association with coinfections. The HIV virus weakens the immune system, increasing vulnerability to exogenous pathogens.^237^ These coinfections, such as CMV, Epstein-Barr virus, tuberculosis, hepatitis C and B, and human papillomavirus, may promote the immune dysregulation caused by HIV, even after initiation of ART, creating an environment conducive to several inflammation-associated complications including CVD (Figure 3).^239^

### Cytomegalovirus

CMV is a large herpes DNA virus that infects vascular endothelial cells.^240,241^ It induces memory CMV-specific T cells that increase with age irrespective of HIV status.^242–244^ Among octogenarians, CMV IgG seropositivity in the general population has been associated with increased mortality from CVD.^245^ In PLWH, CMV seropositivity has been associated with an increased risk of non-AIDS–defining events and CVD/cerebrovascular diseases.^246^ Furthermore, high CMV antibody titers have been associated with increased carotid intimal thickening in people without HIV.^247–250^ Similarly, in PLWH, CMV IgG has been associated with reduced carotid artery distensibility and carotid artery lesions.^251^

In addition to CMV antibody titers, T-cell immune responses against CMV have also been associated with CVD outcomes in PLWH. PLWH mount inflated T-cell responses against CMV with up to 20% of CD4^+^ T cells against a single CMV epitope in the absence of CMV viremia.^29^ CD8^+^ T-cell expansion has also been shown with HIV/CMV coinfection.^252^ CMV-specific cells express the chemokine receptor, CX3CR1, which traffics immune cells to inflamed endothelium. CX3CR1 is more highly expressed on CD4^+^ and CD8^+^ T cells in PLWH compared with people without HIV.^77^ CX3CR1^+^ T cells are important in CVD because they can induce fractalkine expression on endothelial cells,^253^ establishing a positive feedback loop that mediates adhesion and migration of CX3CR1 expressing lymphocytes into the vascular intima.^254,255^ CX3CR1^+^ GPR56^+^ CD57^+^ CD4^+^ T cells (CGC^+^ CD4^+^ T cells) have been shown to be frequently CMV-specific and have been associated with subclinical atherosclerosis in PLWH.^256,257^ GPR56 is a surface marker of cytotoxic T cells.^258^ It is a G-coupled protein receptor expressed by cytotoxic CD4^+^ T cells, whereas CD57 is a marker of late activation or senescence.^258,259^ Higher CX3CR1^+^ CD4^+^ T cells and CMV-specific CD8^+^ T cells have been associated with greater carotid intima thickness^204,205^ possibly due to increased vascular homing.^260^

Initial studies suggested that treating CMV with antiviral therapies could serve as an important CVD risk reduction intervention if CMV is a risk factor for atherosclerosis. A small randomized clinical trial with 30 PLWH treated with valganciclovir (n=14) or placebo (n=16) for 8 weeks showed a significant reduction in CMV DNA in treated participants compared with placebo. This intervention reduced inflammation, which is an important contributor to CVD.^261^ There is a more recent clinical trial by the AIDS Clinical Trials Group (A5383 trial). A study in patients with antineutrophil cytoplasmic antibody–associated vasculitis showed that treatment with valacyclovir reduced CMV reactivation and the proportion of CD28^-^ CD4^+^ T cells.^262^ The reduction in CD28^-^ CD4^+^ T cells was accompanied by improved responses to a pneumococcal vaccine. In a prior study, the same group showed that these cells were also linked to increased arterial stiffness in the same patient population.^263^ Taken together, these studies suggest that future therapies targeting CMV to reduce the anti-CMV T-cell immune response could have a role in the prevention of atherosclerosis progression in CMV-positive HIV(+) and HIV(−) people.

### Hepatitis C Virus

Due to shared transmission routes, HIV/hepatitis C virus (HCV) coinfection is common, with about 20% of PLWH having HCV.^264^ Like HIV, chronic HCV infection is associated with coronary artery disease even after adjusting for traditional risk factors.^265–267^ The risk for CVD is 2.9 higher in HIV/HCV coinfected individuals compared with HIV monoinfected individuals.^268^ Studies suggest a potential mechanism involving higher TF activity and associated CD4^+^ T-cell activation in HCV patients, highlighting the complex relationship between immune activation, microbial translocation, coagulopathy, and CVD.^269^ In a study assessing immune activation in HIV/HCV coinfection, PLWH, chronic hepatitis, and cirrhosis had elevated levels of serum sCD14 and IL-6 alongside incomplete CD4 restoration. While the percentage of activated CD4^+^ and CD8^+^ T cells and Treg was higher in HIV-infected patients than in healthy controls, no significant differences were noted among different HIV patient groups.^270^ In a separate study, inflammation biomarkers, including IFN-α, were found to be higher in individuals coinfected with HCV and HIV compared with those with HCV alone. Some of these biomarkers decreased following successful sustained virological suppression of HCV.^271^ Successful sustained virological suppression of HCV may decrease inflammation and immune activation, potentially mitigating the risk of cardiovascular events.^272^

### Tuberculosis

PLWH with active and latent tuberculosis have increased levels of immune activation, elevated plasma soluble biomarkers of inflammation, such as sCD14, and increased T-cell activation.^273–277^ These elevated biomarkers are indicative of the progression of HIV disease and are associated with a higher risk of mortality.^278,279^

PLWH with latent tuberculosis have a higher likelihood of converting to active tuberculosis.^275^ In a cohort of African adults living with HIV on ART, latent tuberculosis was associated with immune dysregulation and an increase in markers of inflammation (TNF-α, IL-6, IL-12p70, IL-15, and IL-17A).^276^ Some of these markers, such as IL-17A and TNF-α, may promote vascular and endothelial dysfunction, leading to the development and progression of CVD.^167,194,280–282^

### Herpes Simplex Virus 2

Herpes simplex virus 2 (HSV-2) coinfection may act as a cofactor for increased HIV replication, and the use of acyclovir/valacyclovir to target HSV-2 may have localized benefits on HSV-2 with mucosal level HIV suppression and decreased monocyte activation.^283,284^ In a study of 291 men living with HIV in the MACS, infection with multiple herpes viruses was found to be associated with elevated coronary arterial calcium. However, HSV-2 seropositivity in this cohort had the strongest association with subclinical atherosclerosis, with a 4-fold increased risk.^285^ Note that in a different population-based cross-sectional study not specific to HIV, they found that HSV-2 was positively associated with CVD.^286^

### Kaposi Sarcoma and HHV8

Human herpesvirus 8 (HHV8) is implicated in the development and progression of atherosclerosis. Support for this hypothesis is derived from proatherogenic characteristics observed in in vitro studies on the vessel wall.^287^ Additionally, a postmortem report of AIDS patients revealed a higher incidence of macroscopic atheromatous lesions in individuals with Kaposi sarcoma, providing further evidence for the association between the virus and atherogenesis.^288^ Vascular endothelial cells infected with HHV8 demonstrate the ability to stimulate the generation of growth factors, leading to angiogenesis, increased proliferation of endothelial cells, heightened vascular permeability, and cytokine production.^288^

The prevalence of HHV8 seropositivity among PLWH varies geographically, with the highest percentages reported among men who have sex with men.^289–291^ HHV8 seropositivity is associated with higher levels of CRP and T-cell activation, indicating its potential role in chronic immune activation in chronically treated patients with HIV.^292^ In a study involving 141 virologically suppressed PLWH on ART, coinfection with HSV-2 and HHV8 was associated with an increase in hs-CRP and progression of CIMT. Notably, in this study, HHV8 exhibited the strongest correlation with the progression of CIMT.^293^ A separate retrospective study of postmortem samples from PLWH had ≈3.4× more likely to have macroscopic atheroma than the Kaposi sarcoma negative group. Murine studies of APOE^−/−^ mice on a high-fat diet infected with viruses such as HHV8 (MHV-68) had accelerated atherosclerosis, which further supports a possible role for HHV8 in accelerating atherosclerosis in the presence of other CVD risk factors.^287^

## IMPACT OF ART ON CVD

ART has significantly improved the prognosis and quality of life for PLWH. Early ART initiation helps suppress viral replication and restore immune function.^294^ One of the primary objectives of ART is to suppress viral replication, allowing the immune system to recover. ART initiation at an early stage leads to a substantial reduction in viral load, enabling the restoration of CD4^+^ T-cell counts, which are crucial for immune function.^3,295^ Studies have shown an increased risk of CVD in PLWH independent of traditional risk factors,^21,296^ and some ART have been implicated in this increased risk.^297–299^ ART can improve endothelial function and reduce biomarkers of cardiovascular risk, but certain therapies, such as abacavir, were proposed to increase inflammatory markers and risk of myocardial infarction. However, controversy persists as the comparator drugs of that era, which are no longer used, are known to have cardioprotective effects.^60,294^ Combined ART has been reported to have approximately a 26% relative rise in the rate of myocardial infarction in PLWH for each year of exposure within the initial 4 to 6 years of usage.^297^ ART has also been associated with an increase in metabolic syndrome, a known risk of CVD among PLWH.^288^

### Older ART

Most of the drugs used to treat HIV in this early ART era had either unfavorable or favorable off-target (ie, non-HIV related) effects, confounding the interpretation of these newly emerging clinical manifestations. Broadly, some first-generation protease inhibitors (PIs) were associated with visceral obesity (eg, Crixivan Crix belly) and adverse lipid profiles^14^ The thymidine analogs zidovudine and stavudine induced adipocyte-specific mitochondrial toxicity, impairing the ability of adipose tissue to trap free fatty acids.^15,300^ Tenofovir is associated with bone and renal toxicity but favorable lipids and relative CVD protection.^301^ Efavirenz is associated with anorexia and weight loss, which may mitigate the emergence of metabolic syndrome.^302^ These drugs have now been replaced by drugs better designed to only bind their target HIV protein without off-target binding to any host proteins. For example, tenofovir alafenamide (TAF) has replaced tenofovir, which does not have bone or renal toxicity or CVD protective effects.^301^

First generations of antiretroviral drugs, particularly PIs and certain nonnucleoside reverse transcriptase inhibitors, were associated with metabolic complications, including dyslipidemia.^298,303^ PIs, such as indinavir and lopinavir, are notorious for elevating total cholesterol, LDL cholesterol, and triglyceride levels and are implicated in increased progression of subclinical atherosclerosis.^304,305^ To promote optimal virological response, PIs require boosting with ritonavir. Ritonavir is, however, associated with increased plasma lipids, insulin resistance,^68^ endothelial dysfunction,^306^ and macrophage accumulation of cholesterol.^304^ Switching from boosted PIs to raltegravir or dolutegravir results in a better plasma lipid profile and changes in biomarkers associated with cardiovascular events than continuing boosted PI.^294,307^ Older NRTIs, such as stavudine and zidovudine, were also linked to lipid abnormalities. Patients on stavudine were reported to develop a syndrome of short-term fat wasting associated with increased plasma-free fatty acid and triglyceride levels and increased daily fat intake without insulin resistance.^308,309^

PLWH previously exposed to thymidine analogs and didanosine may experience persisting changes in the distribution of visceral adipose tissue and subcutaneous adipose tissue for many years after discontinuation of therapy.^310^ In the visceral compartment, thymidine analogs have been associated with increased mitochondrial toxicity in rats, thus affecting adipocyte distribution and leading to pathological adipose accumulation.^311^ This increases the risk of developing conditions such as hypertension, hypercholesterolemia, and low levels of HDL even years after stopping the treatment.^310^

### Newer Therapies

Newer antiretroviral drugs with improved side effect profiles are available with integrase strand transfer inhibitors gaining prominence. Second-generation integrase strand transfer inhibitors, such as dolutegravir, elvitegravir, and bictegravir, have been well-tolerated and effective in suppressing viral replication. However, an emerging concern has been the association between newer therapies, particularly dolutegravir, and weight gain. Mechanisms of weight gain are poorly understood. However, it has been hypothesized that dolutegravir can directly affect adipocytes, leading to adipogenesis, lipogenesis, oxidative stress, fibrosis, and insulin resistance.^312^ In recent trials, more significant weight gain has been observed in dolutegravir groups compared with efavirenz.^302,313,314^ Some evidence suggests that the greater weight gain observed in people taking dolutegravir-based ART compared with those on efavirenz-based ART might be linked to impaired weight gain in individuals with certain genetic traits, that is, loss of polymorphism in cytochrome P450 2B6 enzyme (CYP2B6; CYP2B6 intermediate or slow metabolizer genotypes) as the CYP2B6 primarily metabolizes efavirenz. These individuals likely experienced slow metabolism of efavirenz, leading to toxicity, rather than dolutegravir causing changes in appetite or metabolism.^315^ Weight gain appears to be most closely related to the antiviral potency of the regimen, and there is no good mechanistic evidence to date that weight gain relates to any off-target effect of any drug or drug class.^302^

Switching from tenofovir disoproxil fumarate (TDF) to TAF has also been associated with significant weight gain in PLWH.^316,317^ One of the proposed mechanisms of weight gain is due to the reduction of plasma HIV RNA levels and mitigating the catabolic effects associated with viremia; therefore, a restorative process leading to improved health occurs. As a result, some individuals experience weight gain that exceeds normal, healthy levels.^318,319^ It is unclear whether TAF independently contributes to weight gain or does not suppress dolutegravir-induced weight gain as TDF does. Although the long-term implications of ART-associated weight gain are not fully understood, limited evidence suggests an increased incidence of hyperglycemia, hypertension, and metabolic syndrome, coupled with an increased risk of cardiovascular complications.^320^

Another shift in HIV practice has been the immediate rather than deferred initiation of ART after diagnosis based on studies such as Strategic Timing of Antiretroviral Treatment (START) (nadir CD4>600 cells/μL), which showed reduced serious AIDS and non-AIDS events in those treated early.^321^ In contrast, the SMART study (nadir CD4 <250 cells/μL) found a 70% decrease in the risk of cardiovascular events in those randomized to early rather than deferred or interrupted ART.^322^ This increased risk correlated with increased proinflammatory markers in the deferred or interrupted group.^4^

## TARGETING THE IMMUNE SYSTEM: BEFORE AND AFTER THE REPRIEVE TRIAL

### Low-Dose Methotrexate

Methotrexate, a competitive dihydrofolate reductase inhibitor known for modifying rheumatoid arthritis progression, has shown promise in reducing mortality among PLWH.^323^ The CIRT (Cardiovascular Inflammation Reduction Trial) was designed to assess whether low-dose methotrexate could prevent CVD events in the general population. There was no reduction in inflammation or CVD effects compared with placebo in this study.^324^ Another pilot study (clinical trial number: NCT01949116) that evaluated the safety and impact of low-dose methotrexate on endothelial function in PLWH reported no significant effect on endothelial function or inflammatory biomarkers with low-dose methotrexate. There was, however, a significant decrease in CD8^+^ T cells. This study was associated with more safety events in the methotrexate group (Table 7).^325^

**Table 7.**
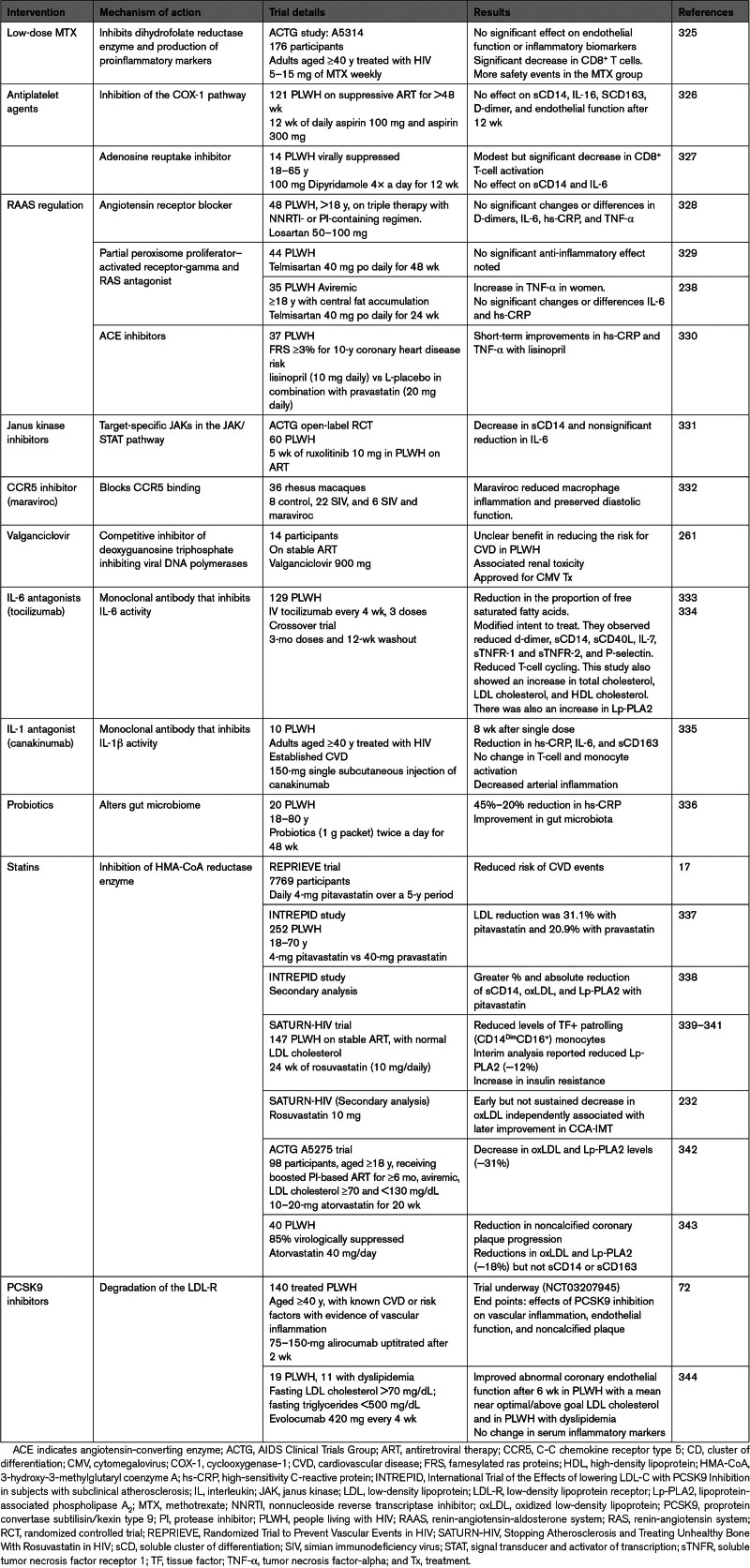
Targeting Immune Activation and Inflammation to Decrease CVD Risk Among PLWH

### IL-1β Antagonists

Canakinumab is a monoclonal antibody against IL-1β that was first shown to reduce inflammation and improve CVD events independent of lipids.^324^ Notably, participants in that study (HIV-negative) received 3 doses of the antibody, and they observed a higher rate of fatal infections than in the placebo group. Among PLWH, a single dose of canakinumab resulted in reduced arterial inflammation measured by fluorodeoxyglucose/positron emission tomography/CT, as well as reduced levels in circulating inflammatory markers, including IL-6 and sCD163. There was no difference in the proportion of activated T cells and monocytes.^335^ IL-1 antagonism has also been shown in smaller studies to improve heart failure through the reduction of systemic inflammatory response.^345,346^

### TNF-α Antagonists

There have been mixed outcomes in the general population with TNF-α antagonists in heart failure trials.^347,348^ No TNF-α has been done among PLWH.

### IL-6 Antagonist

Tocilizumab is a monoclonal antibody against IL-6 and, in a crossover clinical trial among PLWH, has been shown to decrease markers of circulating inflammatory markers, including sCD14, sCD40L, d-dimer, p-selectin, and sTNFR1/2. Notably, there was an increase in sCD163, but this was not significant. Like a previous study among people without HIV, there was an increase in LDL cholesterol, HDL cholesterol, and Lp-PLA2 (lipoprotein-associated phospholipase A_2_).^334^ The increase in lipid classes was not altered by statin treatment. Clinically, there was no improvement in endothelial function as measured by brachial artery flow–mediated dilation.^334^

### Antiplatelet Agents

Aspirin, a traditional cardiovascular prevention treatment, has shown promise in reducing immune activation in PLWH on ART. Aspirin inhibits cyclooxygenase, which is in the pathway that leads to the formation of prostaglandins, downstream of which is the induction of inflammatory cytokines, including IL-6 and IL-1β.^349^ One study observed that low-dose aspirin attenuated sCD14 and activated T cells in ART-treated PLWH.^350^ A longer 12-week randomized controlled trial showed no effects of high or low-dose aspirin on monocyte activation (sCD14 and sCD163), proatherogenic monocyte subsets, T-cell activation, and endothelial dysfunction.^326^ Ongoing trials are exploring aspirin’s immune and vascular effects in PLWH. However, the potential benefits of primary prevention must be weighed against bleeding risks.

### RAAS Regulation

Drugs targeting the RAAS (renin-angiotensin-aldosterone system) may also reduce CVD events by mitigating inflammation and tissue fibrosis pathways. Limited trials have explored the effects of lisinopril and telmisartan in PLWH subjects, showing potential benefits in reducing visceral adiposity, hs-CRP, and TNF-α.^238,330^ No adipocyte fibrosis reduction is seen in telmisartan therapy in addition to ART.^329^ A large retrospective study by Klein et al^351^ indicated a declining risk of myocardial infarction over time in HIV-infected individuals, correlating with increased prescriptions for lipid-lowering and antihypertensive treatments. One study on the impact of losartan on inflammation and tissue fibrosis found that losartan had no effect on carotid intima thickness, T-cell activation, markers of inflammation, or monocyte differentiation in PLWH on ART.^328^

### Omega-3 Fatty Acids

Omega-3 fatty acids, while modestly improving systemic inflammation in PLWH by decreasing levels of IL-6 and TNF-α, do not seem to significantly affect endothelial function based on one small study.^352^

### Statins

Statins, known for their lipid-lowering and potential anti-inflammatory properties, have shown promise in preventing CVD in PLWH.^353^ Studies on atorvastatin and rosuvastatin demonstrated reductions in noncalcified plaque volume, high-risk coronary plaque features, and slowed progression of CIMT.^339^ The Study to Assess the Tolerability and Anti-Retroviral Activity of MK-0518 (SATURN-HIV) trial further highlighted the role of statins in reducing T-cell activation and innate immune activation markers.^339^ The large REPRIEVE focused on the use of a statin, pitavastatin, to reduce cardiovascular events in PLWH. While the primary end point of the trial was CVD prevention, substudies within REPRIEVE explored the impact of pitavastatin on immune activation and reported a reduced risk of a major adverse cardiovascular event compared with those who received a placebo.^17^ A small randomized, double-blind study on the effects of pitavastatin on atherosclerotic-associated inflammatory biomarkers when administered with PI ritonavir-boosted atazanavir in PLWH with dyslipidemia demonstrated that pitavastatin lowered the proportion of activated T cells (HLA-DR^+^ CD38^−^CD4^+^) and programmed cell death-protein PD1^+^ CD4^+^ T cells, thus reducing immune activation.^354^ There was also an increase in basic fibroblast growth factor, which has atherosclerotic-protective effects.

### PCSK9 Inhibitors

PCSK9 (proprotein convertase subtilisin/kexin type 9) plays a vital role in cholesterol metabolism through modulation of the expression of LDL receptors, which bind and remove LDL cholesterol^355^ and predict future cardiovascular risk.^356^ PLWH with or without dyslipidemia have been reported to have elevated levels of PCSK9^71^ associated with foam cell formation and endothelial dysfunction in these individuals.^71^ Leucker et al^357^ showed that rapid improvement in coronary endothelial function in PLWH treated with PCSK9 inhibitor was associated with reduced Lp(a) (lipoprotein[a]) and not LDL cholesterol. PCSK9 antibodies (PCSK9i) have also shown effectiveness in reducing cardiovascular events in individuals with coronary artery disease who are intolerant to statin therapy.^358^ In a small study involving 11 PLWH with dyslipidemia not diagnosed with coronary artery disease and 19 PLWH with nearly optimal or above goal low-density lipoprotein cholesterol levels, the inhibition of PCSK9 with evolocumab demonstrated an improvement in abnormal coronary endothelial function.^344^ In this study, no improvement was reported in serum inflammation biomarkers. Another study is underway and will shed more light on the effects of PCSK9 inhibition with alirocumab on vascular inflammation, endothelial function, and noncalcified plaque in ART-treated participants.^72^

## CONCLUSION AND FUTURE DIRECTION

The persistent dysregulation of both adaptive and innate immunity in chronic HIV infection, in the presence of clinically suppressed viral load through effective ART, underscores the complexity of immune activation dynamics. ART reduces but does not normalize immune activation. While the specific factors that drive heightened immune activity may vary among individuals, the resultant systemic inflammation significantly contributes to the amplification of well-established proatherogenic mechanisms. This intricate interplay between chronic HIV infection, immune activation, and ASCVD emphasizes the need for targeted therapeutic approaches. Epidemiological and mechanistic research has enhanced our understanding of the extent and drivers of CVD in the general population and among PLWH. Nevertheless, several gaps in the literature need to be investigated further to help improve patient outcomes (Table 8).

**Table 8.**
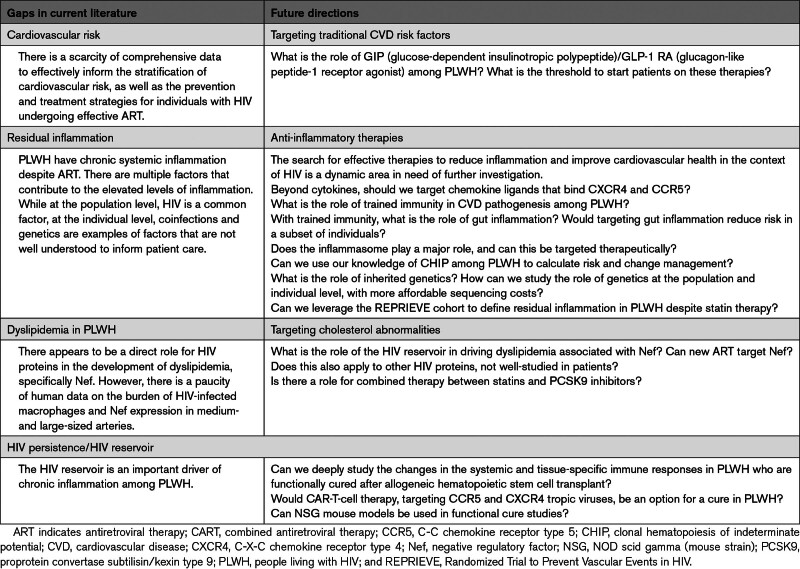
Gaps in Literature and Future Directions

## ARTICLE INFORMATION

### Acknowledgments

Images were created using BioRender.

### Sources of Funding

L.M. Obare was supported by the Gilead HIV Scholars Research Program. T. Temu was supported by grant K01HL147723. S.A. Mallal was supported by the Tennessee Center for AIDS Research grant P30 AI110527. C.N. Wanjalla was supported by the Doris Duke CSDA 2021193 (grant K23 HL156759), the Burroughs Wellcome Fund grant 1021480, and the Gilead HIV Scholars Research Program.

### Disclosures

None.
